# Molecular evidence for the involvement of a polygalacturonase-inhibiting protein, GhPGIP1, in enhanced resistance to Verticillium and Fusarium wilts in cotton

**DOI:** 10.1038/srep39840

**Published:** 2017-01-12

**Authors:** Nana Liu, Xueyan Zhang, Yun Sun, Ping Wang, Xiancai Li, Yakun Pei, Fuguang Li, Yuxia Hou

**Affiliations:** 1 College of Science, China Agricultural University, No. 2 Yuanmingyuan West Road, Beijing 100193, People’s Republic of China; 2 State Key Laboratory of Cotton Biology, Institute of Cotton Research of the Chinese Academy of Agricultural Sciences, Anyang 455000, People’s Republic of China

## Abstract

Polygalacturonase-inhibiting protein (PGIP), belonging to a group of plant defence proteins, specifically inhibits endopolygalacturonases secreted by pathogens. Herein, we showed that purified GhPGIP1 is a functional inhibitor of *Verticillium dahliae* and *Fusarium oxysporum* f. sp. *vasinfectum*, the two fungal pathogens causing cotton wilt. Transcription of *GhPGIP1* was increased in cotton upon infection, wounding, and treatment with defence hormone and H_2_O_2_. Resistance by GhPGIP1 was examined by its virus-induced gene silencing in cotton and overexpression in *Arabidopsis*. GhPGIP1-silenced cotton was highly susceptible to the infections. GhPGIP1 overexpression in transgenic *Arabidopsis* conferred resistance to the infection, accompanied by enhanced expression of pathogenesis-related proteins (PRs), isochorismate synthase 1 (*ICS1*), enhanced disease susceptibility 1 (*EDS1*), and phytoalexin-deficient 4 (*PAD4*) genes. Transmission electron microscopy revealed cell wall alteration and cell disintegration in plants inoculated with polygalacturonase (PGs), implying its role in damaging the cell wall. Docking studies showed that GhPGIP1 interacted strongly with C-terminal of *V. dahliae* PG1 (VdPG1) beyond the active site but weakly interacted with C-terminal of *F. oxysporum* f. sp. *vasinfectum* (FovPG1). These findings will contribute towards the understanding of the roles of PGIPs and in screening potential combat proteins with novel recognition specificities against evolving pathogenic factors for countering pathogen invasion.

Plant cell wall is rich in polysaccharides and offers the first barrier against pathogens, which needs to be broken for the infection to set in. Pathogens secrete several enzymes that can decompose the cell wall. Among these, polygalacturonases [α-1,4-D-polygalacturonases (EC 3.21.15), PGs] are the most important enzymes that hydrolyse polygalacturonan, a cell wall component, into oligosaccharides, thus breaking the cell wall and providing nutrition for further infection of the pathogens^1^. PGs are considered to be pathogenic factors in several microorganisms such as fungal pathogens *Botrytis cinerea*^2^, *Alternaria citri*^3^, and *Claviceps purpurea*^4^ as well as bacterial pathogens *Agrobacterium tumefaciens*^5^ and *Ralstonia solanacearum*^6^.

Polygalacturonase-inhibiting protein (PGIP) is a cell wall-binding protein that effectively and specifically binds with PGs and inhibits further invasion of pathogens^7^. PGIP belongs to leucine-rich repeat (LRR) super family, members of which contain ~20–30 amino acid (LxxLxLxxNxL or LxxLxLxxCxxL, where, L = I, L, V, F; N = N, T, S, C; C = C, S; x = any amino acid) repeat sequences^8^. The repeats (generally 10) form β-sheet/β-turn/β-sheet/α-helix containing LRR motifs, which are considered as protein–protein interaction areas^9^. This is the reason why many PGIPs have conserved sequences^10^. PGIPs interact with the active site amino acid residues of PGs through the amino acid exposed outside of the LRR motif, thereby, inhibiting their activity^11^,^12^,^13^. In plants, PGIP gene clusters are usually present in specific chromosomal regions and form a small gene family. In kidney beans, for example, at least five PGIP genes are located on chromosome 10 over an area of 165 kb^14^. In *Arabidopsis thaliana*, PGIP genes, *AtPGIP1* (At5g06860) and *AtPGIP2* (At5g06870), with 76.1% sequence identity at the amino acid-level, are present in tandem on chromosome 5, with their coding sequences separated only by a stretch of 507 bp^15^. However, there are exceptions; for example, *Brassica napus* genes, *BnPGIP1* and *BnPGIP2*, with 67.4% sequence identity at the amino acid level, are located on different chromosomes^16^. The transgenic expression of pear PGIP in tomato was shown to limit the colonization by *B. cinerea*^17^ in transgenic tobacco leaves. Similarly, transgenic tobacco plants expressing *Capsicum annuum* CaPGIP1 exhibited increased resistance to PGs from *Alternaria alternata* and *Colletotrichum nicotianae*^18^, and the overexpression of *AtPGIPs* in *A. thaliana* enhanced its resistance to *B. cinerea* infection^15^, indicating that the over-expression of PGIP could effectively inhibit the infection by various fungal pathogens.

The expression of PGIP can be induced by several biotic and abiotic stress factors. The biological inducers mainly include fungi and insects and non-biological inducers include wounding, salicylic acid (SA), low temperature, salt, methyl jasmonate (MeJA), oligomeric galacturonic acid, etc^19^. In *Arabidopsis*, the expression of *AtPGIP1* and *AtPGIP2* was up-regulated after inoculation with *B. cinerea*, albeit using different signal transduction pathways. The expression of *AtPGIP2* was induced by MeJA whereas that of *AtPGIP1* increased sharply after treatment with oligogalacturonic acid, remaining unaffected by SA, MeJA, and ethylene. In addition, low temperature could induce the expression of *AtPGIP1* but had no effect on *AtPGIP2* expression^15^. In *Phaseolus vulgaris, PvPGIP2* could be induced by all the above-mentioned treatments, whereas *PvPGIP3* was induced in suspension culture cells treated with oligogalacturonic acid, but not dextran. However, *PvPGIP4* did not express under any of these treatments^20^. Overall, different PGIP genes express through different signal transduction pathways.

Virus-induced gene silencing (VIGS), as a tool for loss of gene function analysis, was developed through *Agrobacterium tumefaciens*-mediated transient assays^21^. Seedlings or roots are inoculated or sprayed with *Agrobacterium* culture carrying a viral vector, containing the gene of interest, to disrupt the endogenous genes^22^,^23^. Using VIGS technology, *Capsicum annuum* PGIP (CaPGIP)-silenced pepper plants infected with *Phytophthora capsici* were observed to have decreased transcript levels of *CaPGIP1, CaPGIP2*, and *CaPGIP3* and enhanced sensitivity to *P. capsici* infection compared to the plants infected with a control vector^18^. This report suggested that PGIP genes function in systemic resistance of plants to pathogens.

Cotton is an important cash crop worldwide, and is a significant source of feed and fibre. To increase its yield and quality, several strategies such as molecular breeding, genetic engineering of new cultivars, and disease control have been attempted. Genetic engineering of novel genes into cotton is a robust approach for genome improvement^21^.

Cotton Verticillium wilt, called the cancer of cotton, caused by *Verticillium dahliae* Kleb, has been one of the main impediments in sustainable cotton production in recent years. It is a soil- and seed-borne vascular disease, and is difficult to control. Moreover, the host range of *V. dahliae* is wide^24^. Upon infection, *V. dahliae* produces a variety of cell wall degradation enzymes such as pectinases and cellulases. These enzymes can degrade the cell wall of the host plant, aiding the colonization, spread, and extension of the pathogen^25^,^26^,^27^. Of all these enzymes, pectinases such as polygalacturonase, pectate lyase, and pectin esterase are the most widely studied. Carder^28^,^29^ showed that pectinase activity and Verticillium wilt pathogen toxicity were positively correlated; high virulent strain of Verticillium wilt demonstrated higher pectinase activity, whereas low virulence isolates produced almost no pectinase. Therefore, pectinase was perceived as the dominant biochemical factor of *V. dahliae* virulence. Studies have also shown that cellulase production in *V. dahliae* positively correlates with its invasion^30^. These reports suggested that cell wall degrading enzymes play an important role in the virulence of *V. dahliae*. PGIP can selectively inhibit a fungal pectinase called endopolygalacturonase. It promotes the accumulation of oligogalacturonic acid, effectively blocking the further course of fungal infection and inhibiting the occurrence of the respective diseases^31^. PGIP is mainly located in the cell wall and endomembrane system, having PG inhibitory activity that is positively related to disease resistance in plants^32^. Therefore, it is imperative to understand the expression and function of PGIP gene before introducing it into cotton plants for enhancing their resistance to *V. dahliae* and other pathogens.

The cotton-wilt Fusarium is a disease of cotton, worldwide. The causal pathogen secretes several kinds of cell wall-degrading enzymes during root penetration and host plant colonization, including PGs, which play a major role in infection^33^,^34^. The pathogenic mechanisms and the induction of the wilt symptom by the fungus are poorly understand, although endopolygalacturonases are considered to be involved in the process^35^.

In the present study, the gene encoding cotton PGIP, *GhPGIP1*, was isolated and expressed in *Escherichia coli*. We demonstrated the effectiveness of purified GhPGIP1 against two PGs from *V. dahliae* and *Fusarium oxysporum* f. sp. *vasinfectum*. Using *in silico* molecular docking, we elucidated the structural basis for interaction between GhPGIP1 and the PGs and the effectiveness of such interaction in the inhibition of the PGs. We also explored the potential of GhPGIP1 in reinforcement of the cell wall barrier for enhancing the resistance of cotton to the fungal pathogens. Furthermore, the resistance offered to these pathogens by *GhPGIP1* was examined using its virus-induced gene silencing in cotton and overexpression in *Arabidopsis*.

## Results

### Cloning and characterization of *GhPGIP1*

The full length cDNA of *GhPGIP1* (GenBank Accession No. KR108279) contained 1193 bp, had an ORF of 990 bp, and was predicted to encode a protein of 330 amino acids with a theoretical molecular mass of 34.5 kDa and pI of 8.5 (Supplementary Fig. 1).

**Figure 1:**
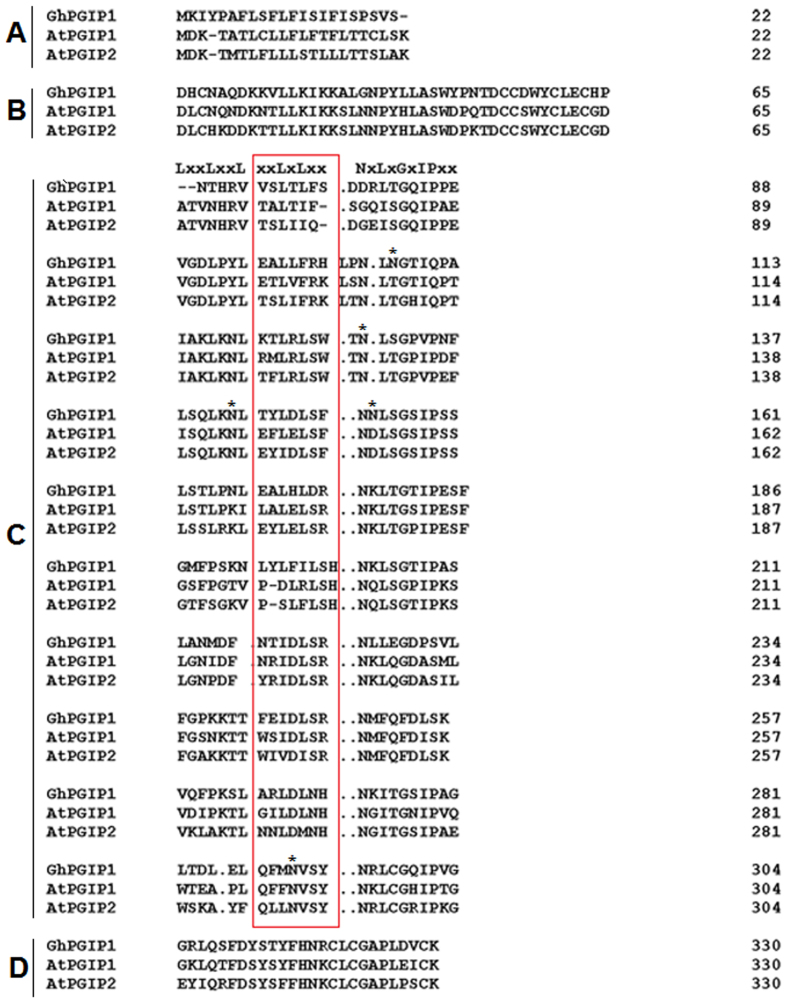
Comparison of the predicted amino acid sequences of GhPGIP1 with AtPGIP1 and AtPGIP2 from *Arabidopsis*. The predicted amino acid sequences were aligned using Clustal Omega and the following typical PGIP domains were identified: **(A)** signal peptide, **(B)** putative N-terminus of PGIP protein, **(C)** 10 conserved LRR sequences, and **(D)** C-terminus. The central domain of GhPGIP1 was detected and contained consensus sequence characteristics for PGIPs, xxL xLxx. NxLx. GxIPxxLxxL.xxL. The boxed region represents a β-sheet/β-turn motif (xxLxLxx). The dots indicate the gaps in the aligned sequences of the LRR modules.

**Figure 2:**
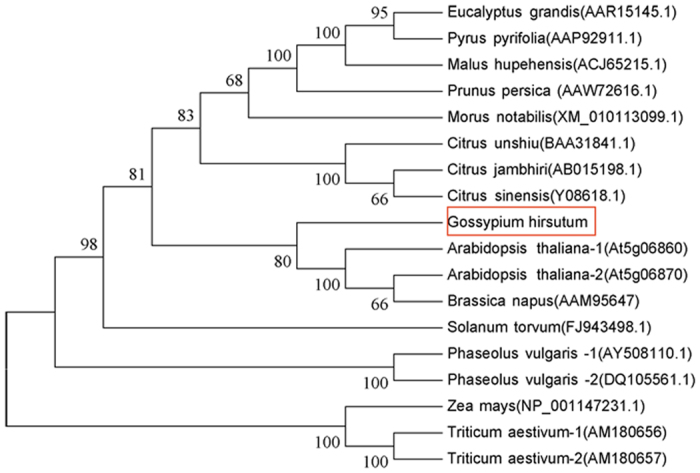
Phylogenetic tree showing the close relationship of GhPGIP1 with other known monocot and dicot PGIPs. The deduced amino acid sequences of PGIPs retrieved from GenBank were aligned with Clustal W using MEGA5.1 and a neighbour-joining tree was built based on the alignment. The position of GhPGIP1 is emphasized in the red box. GenBank accession numbers of each PGIP are listed in the brackets behind the species names.

The predicted protein displayed the typical topology of previously described PGIPs, which included a 22-amino acid signal peptide for secretion (domain A), an N-terminal domain (domain B), an LRR domain composed of 10 imperfect modules characterized by an extracytoplasmic type LRR consensus sequence (domain C), and a C-terminal domain (domain D; Fig. 1). Phylogenetic analysis of GhPGIP1 was performed by constructing a phylogenetic tree that consisted of several main branches (Fig. 2), in which cotton PGIP was observed to have a high degree (80%) of amino acid identity with PGIPs from *Arabidopsis*^15^, suggesting that they might have similar features and functions.

### *GhPGIP1* transcripts accumulate in response to stress factors

**Figure 3:**
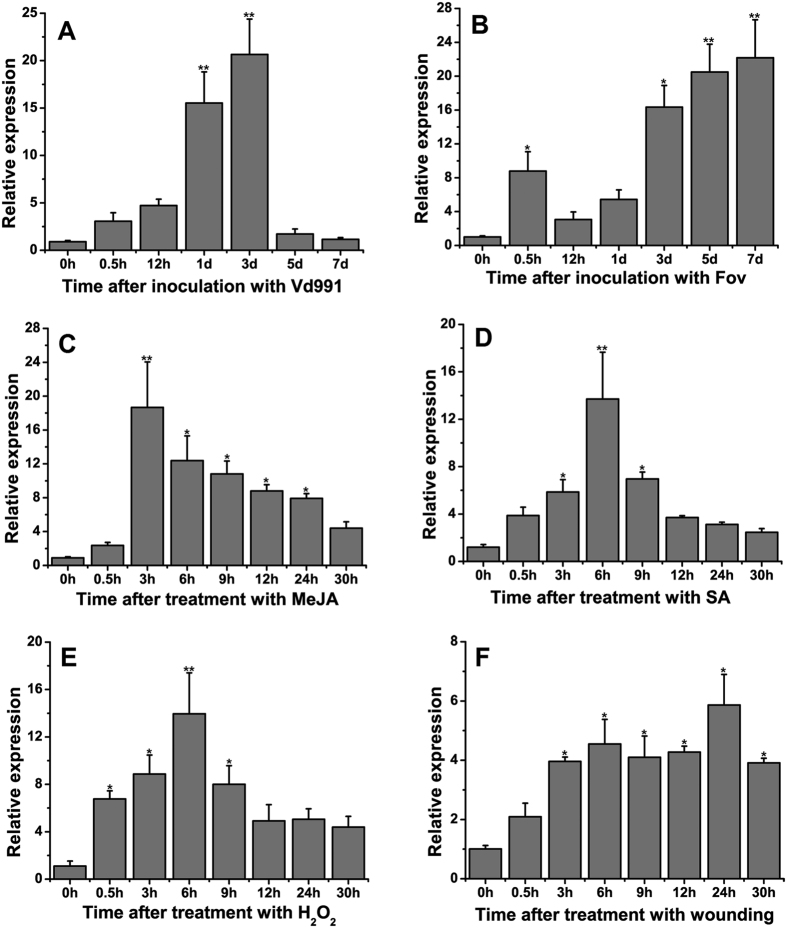
Expression analysis of GhPGIP1 by qRT-PCR. **(A)** Expression of GhPGIP1 in cotton seedlings inoculated with Vd991 at 0, 0.5, and 12 h, and 1, 3, 5, and 7 d of infection. **(B)** Expression of GhPGIP1 in cotton seedlings inoculated with *Fusarium* f. sp. *vasinfectum* at 0, 0.5, and 12 h, and 1, 3, 5, and 7 d of infection. **(C)** Expression of GhPGIP1 after 0, 0.5, 3, 6, 9, 12, 24, and 30 h of MeJA induction. **(D)** Expression of GhPGIP1 after 0, 0.5, 3, 6, 9, 12, 24, and 30 h of SA induction. **(E)** Expression of GhPGIP1 after 0, 0.5, 3, 6, 9, 12, 24, and 30 h of H_2_O_2_ induction. **(F)** Expression of GhPGIP1 after 0, 0.5, 3, 6, 9, 12, 24, and 30 h of wound induction. For MeJA, SA, H_2_O_2_, and wound induction, the samples after inoculation with double distilled water were used as control; however, for Vd991 and *Fusarium* f. sp. *vasinfectum*, samples treated with czapeks medium were used as control. Data were collected from three independent biological replicates. The data are means ± standard errors (n = 3). Asterisks indicate significant differences compared to the control [least significance differences (LSD), *P < 0.05, ** *P* < 0.01 ].

The expression of *GhPGIP1* under various stresses, such as MeJA, SA, H_2_O_2_, wounding, and inoculation with *V. dahliae* and *F. oxysporum* f. sp. *vasinfectum* was investigated by real-time PCR. Inoculation of cotton seedlings with *V. dahliae* caused an up-regulation of *GhPGIP1* expression for the first 5 d, the expression reaching the highest level on 3 d (Fig. 3A). However, inoculation with *F. oxysporum* f. sp. *vasinfectum* caused a transient increase for the first 0.5 h, which was followed by a continued increase until the seventh day (Fig. 3B). The transcription level in cotton seedlings treated with MeJA increased markedly at 3 h (Fig. 3C) whereas upon SA treatment, *GhPGIP1* levels reached the highest at 6 h (Fig. 3D) and gradually decreased thereafter. Upon H_2_O_2_ treatment, the expression increased until 6 h, reached the maximum (Fig. 3E), and then decreased. On wounding, the transcription increased after 0.5 h and the increase continued until 24 h (Fig. 3F).

### *In vitro* inhibition profile of GhPGIP1

**Figure 4:**
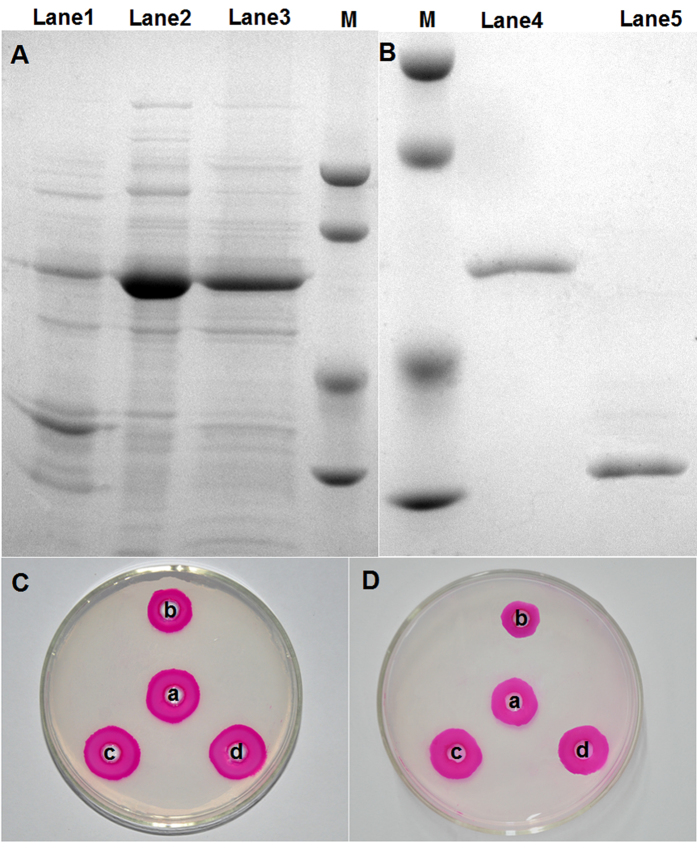
Purification and inhibitory activity of GhPGIP1 against VdPG1 and FovPG1. **(A)** Expression of GhPGIP1 in *E. coli*. Lane 1, total protein expressed in *E. coli* before IPTG induction; Lane 2, total protein extract in the supernatant of *E. coli* expressing the recombinant GhPGIP1, after IPTG induction; Lane 3, total protein extract in the pellet of *E. coli* expressing the recombinant GhPGIP1, after IPTG induction. M, molecular mass marker, 94.0 kDa, 66.2 kDa, 45.0 kDa, 33.0 kDa from top to bottom. **(B)** Purification of GhPGIP1. Lane 4, flow through from a Ni-IDA superflow column; Lane 5, purified GhPGIP1, with fusion tags removed. M, molecular mass marker, 94.0 kDa, 66.2 kDa, 45.0 kDa, 33.0 kDa from top to bottom. **(C)** Agarose diffusion assay of VdPG1. a, 15 μL enzyme; b, 15 μL enzyme + 10 μg GhPGIP1; c, 15 μL enzyme + 15 μL phosphate-buffered saline; d, 15 μL enzyme + 10 μg heat-denatured GhPGIP1. **(D)** Agarose diffusion assay of FovPG1. a, 15 μL enzyme; b, 15 μL enzyme + 10 μg GhPGIP1; c, 15 μL enzyme + 15 μL phosphate-buffered saline; d, 15 μL enzyme + 10 μg heat-denatured GhPGIP1.

The inhibition of two different PGs from *V. dahliae* (VdPG1) and *F. oxysporum* f. sp. *vasinfectum* (FovPG1) by GhPGIP1 was assessed *in vitro.* The purified GhPGIP1 protein was obtained by cleavage of His-tag from His-GhPGIP1 fusion protein using protease digestion with biotinylated thrombin (Fig. 4A,B, Supplementary Fig. 2). The ability to inhibit VdPG1 and FovPG1 was carried out by agarose diffusion assay^36^. The results revealed that GhPGIP1 inhibited both VdPG1 and FovPG1 (Fig. 4C,D), with an IC_50_ of 44.37 and 50.13 μg/mL, respectively (Table 1).

**Figure 5:**
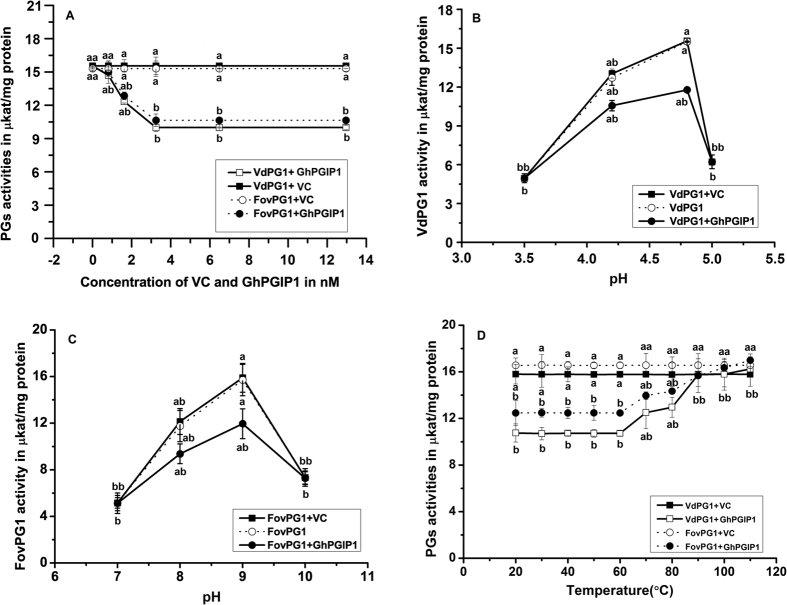
VdPG1 and FovPG1 inhibition assay. **(A)** Inhibition of VdPG1 and FovPG1 at different concentrations of GhPGIP1. VdPG1 (15 ng) and FovPG1 (25 ng) were incubated with GhPGIP1 and vector control (within a range of 0.405–12.96 nM) and the enzyme activity at different concentrations were plotted. **(B)** pH optimum of VdPG1. Reaction of VdPG1 (15 ng) with GhPGIP1 and 3.24 nM vector control was assayed and the enzyme activity was detected over different pH and plotted for determination of the pH optima. **(C)** pH optimum of FovPG1. Reaction of FovPG1 (25 ng) with GhPGIP1 and 3.24 nM vector control was assayed and the enzyme activity was detected over different pH and plotted for the determination of the pH optima. **(D)** Temperature stability of GhPGIP1. Reactions of VdPG1 (15 ng) and FovPG1 (25 ng) with GhPGIP1 and 3.24 nM vector control were assayed over a temperature range of 20–110 °C for 1 h, GhPGIP1 temperature stability was determined by plotting the enzyme activity of the two PGs with temperature. The data points were means of a single experiment performed at least thrice with different batches of enzymes. Results are shown as means ± standard errors. Means marked with the same letter were not significantly different according to Tukey’s HSD test at P < 0.05.

GhPGIP1 displayed a differential activity profile against the two fungal PGs (Fig. 5A); the effects of various parameters on VdPG1 and FovPG1 inhibition are presented below. The VC (vector control) as well as the lowest GhPGIP1 concentration tested did not inhibit either of the enzymes. A gradual increase in inhibition was observed with the increasing inhibitor concentration (Fig. 5A). A significant increase in inhibition was observed at 0.81, 1.62, and 3.24 nM GhPGIP1 concentrations, with inhibition being 5.50%, 20.57%, and 30.69% for VdPG1 and 2.03%, 16.10%, and 27.39% for FovPG1, respectively. However, further increase in GhPGIP1 concentration (up to 6.48 and 12.96 nM) resulted in marginal increase in inhibition. GhPGIP1 at 3.24 nM concentration was used for further studies.

### pH optima of VdPG1 and FovPG1 inhibition and thermal stability of GhPGIP1

Inhibition of VdPG1 by GhPGIP1, assayed over a pH range of 3.5–5.0, revealed that the optimum pH for inhibition was between 4.2 and 4.8, with an inhibition of 26.88 and 31.45%, respectively (Fig. 5B). FovPG1 inhibition by the same concentration of GhPGIP1 over a pH range of 7.0–10.0 showed that the pH optimum for its inhibition was between 8.0 and 9.0, with 23.79% and 29.61% inhibition, respectively (Fig. 5C).

GhPGIP1 (3.24 nM) was equally active from 20 °C to 60 °C (Fig. 5D). The activity dropped significantly to 21.2% at 70 °C and further to 10.5% and 2.2% at 80 °C and 90 °C, respectively. No inhibition of PGs was observed beyond this temperature.

### GhPGIP1 is capable of restricting fungal infection in leaves of transgenic *Arabidopsis* plants by acting as a functional inhibitor

**Figure 6:**
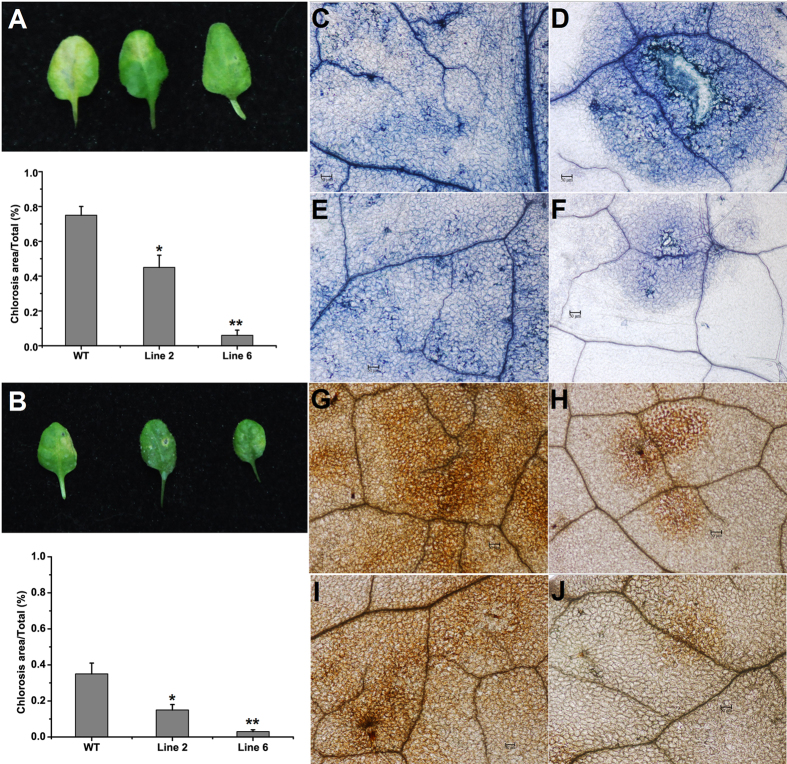
Transgenic *Arabidopsis* plants displaying increased resistance to *V. dahliae* and *Fusarium oxysporum* f. sp. *vasinfectum*. **(A)** Disease symptoms (top panel) and disease lesion area (bottom panel) after inoculation with *V. dahliae* in the wild type and transgenic *Arabidopsis* at 4 days post-inoculation (dpi). **(B)** Disease symptoms (top panel) and disease lesion area (bottom panel) after inoculation with *F. oxysporum* f. sp. *vasinfectum* in the wild type and transgenic *Arabidopsis* at 4 dpi. **(C)** and **(D)** Trypan blue staining of (C) wild-type and (**D**) transgenic *Arabidopsis* leaves, 3 dpi with *V. dahliae*. Bar = 50 μm. **(E)** and **(F)** Trypan blue staining of (**E**) wild type and (**F**) transgenic *Arabidopsis* leaves, 3 dpi with *F. oxysporum* f. sp. *vasinfectum*. Bar = 50 μm. **(G)** and **(H)** DAB staining of (**G**) wild type and (**H**) transgenic *Arabidopsis* leaves, 3 dpi with *V. dahliae*. Bar = 50 μm. **(I)** and **(J)** DAB staining of (**I**) wild-type and (**J**) transgenic *Arabidopsis* leaves 3 dpi with *F. oxysporum* f. sp. *vasinfectum*. Bar = 50 μm. The disease assay was carried out by drop inoculation of *V. dahliae* and *F. oxysporum* f. sp. *vasinfectum* on detached leaves. Data were collected from three independent biological replicates, and at least 10 leaves were used in each biological replicate. The average lesion areas are expressed as means ± standard errors (n = 4). All the data were statistically analysed. Asterisks indicate significant differences compared to the control (Student’s *t*-test, *P < 0.05, **P < 0.01).

To test whether the accumulation of GhPGIP1 in transgenic *Arabidopsis* plants resulted in an increased resistance to tissue degradation caused by the two pathogens, the leaves of line 2 and line 6 (Supplementary Fig. 3) were drop-inoculated with a suspension of *V. dahliae* and *F. oxysporum* f. sp. *vasinfectum* conidia as described previously^37^. The transgenic plants exhibited enhanced resistance to these pathogens (Fig. 6A,B). Notably, leaf etiolation symptoms in the transgenic plants decreased at the inoculation site, particularly in line 6. Trypan blue staining was performed for the infected leaves to observe the fungal growth and disease lesions. The results revealed that *V. dahliae* infection increased the number of dead cells, with larger and expanding cell death areas observed beyond the inoculation site in the wild-type (WT) plants at 72 hours post-infection (hpi; Fig. 6C). However, in transgenic *Arabidopsis* plants, dead cells were restricted to the inoculation site that showed no noticeable spread (Fig. 6D). Similarly, the germination and growth of *F. oxysporum* f. sp. *vasinfectum* was significantly attenuated in the transgenic plants compared to that in the WT plants (Fig. 6E,F). Leaves were also treated with 3,3-diaminobenzidine (DAB), which forms a precipitate in the presence of H_2_O_2_^38^,^39^. After inoculation with *V. dahliae*, less H_2_O_2_ accumulated in the transgenic leaves compared to the WT leaves (Fig. 6G,H). Transgenic lines inoculated with *F. oxysporum* f. sp. *vasinfectum* showed no H_2_O_2_ accumulation at 72 hpi whereas accumulation was detected in infected WT plants and was accompanied with deregulated resistance against the pathogen and cell death (Fig. 6I,J).

### GhPGIP1 is required for expression of genes in the disease-resistance pathway

**Figure 7:**
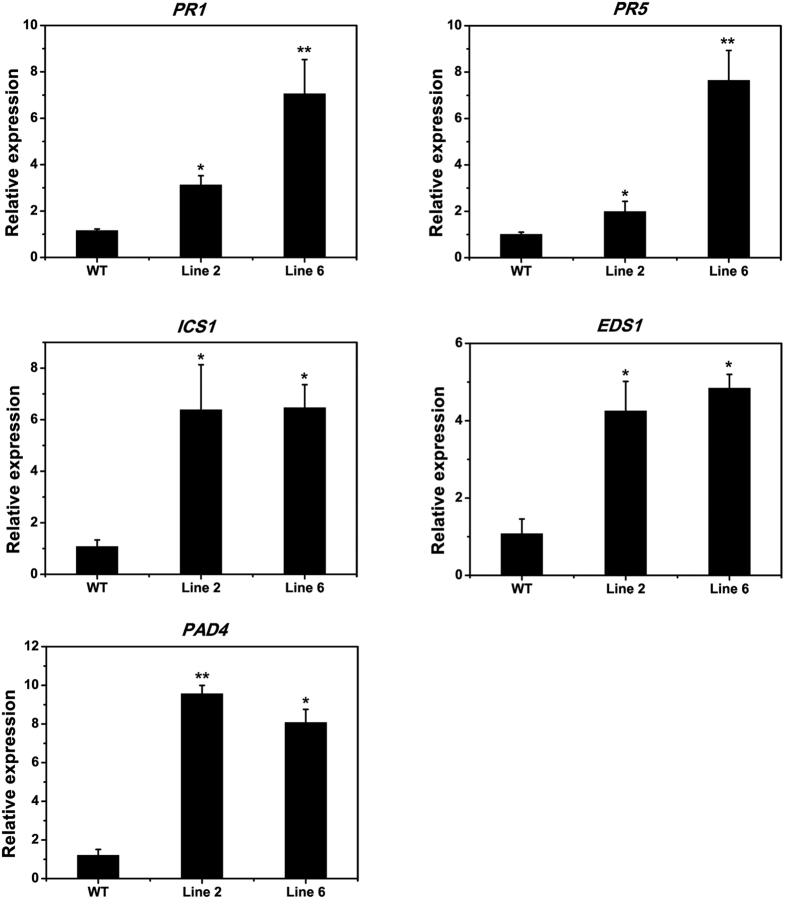
Expression of disease resistance-related genes and plant defensin genes. Total RNAs were extracted from the soil grown WT and transgenic Arabidopsis that inoculation with *V. dahliae* 5-weeks later. The relative transcription levels were detected with Arabidopsis *EF-1-α* as an endogenous reference for normalization. The expression level of WT was set to 1. The experiment occurred with three independent biological replicates, and error bars indicate standard error (n = 3). Significance of difference compared with WT was analysed statistically (Student’s *t*-test, *P < 0.05, **P < 0.01).

To understand the role of GhPGIP1 in the defence response of plants, the expression levels of *Arabidopsis* defence genes in WT and overexpression lines inoculated with *V. dahliae* were detected under normal growth conditions. These genes included the pathogenesis related protein genes *PR1* and *PR5, ICS1, EDS1*, and *PAD4*. In the over expressing lines, expression of some of these genes was significantly up-regulated (Fig. 7). In transgenic line 6, *PR1, PR5, ICS1*, and *EDS1* were up-regulated 6.23 ± 2.21, 7.13 ± 1.33, 6.09 ± 0.98, and 4.88 ± 0.56 times, respectively. In transgenic line 2, *PR1, ICS1, EDS1*, and *PAD4* were significantly up-regulated than in the WT plants. These results suggest that GhPGIP1 directly or indirectly affects the expression of these genes and enhances the resistance of transgenic plants to the pathogens.

### Silencing of *GhPGIP1* by *Agrobacterium*-mediated VIGS

**Figure 8:**
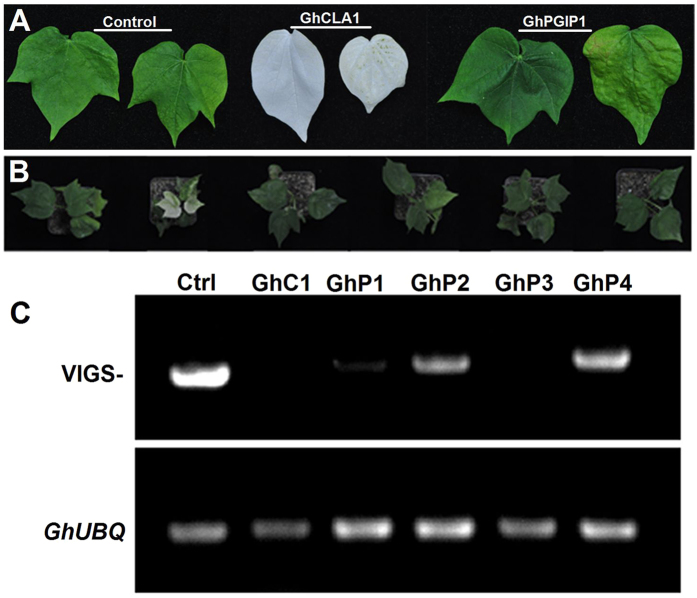
VIGS-mediated gene silencing in cotton. **(A)** *Agrobacterium*-mediated VIGS in cotton leaves. **(B)** The phenotypes of cotton triggered by *GhCLA1* and *GhPGIP1*. The *Agrobacterium* cultures containing pTRV1 and pTRV2: GhCLA1, pTRV2: GhPGIP1 were mixed in a 1:1 ratio and were infiltrated into the fully extended cotyledons of 2-week-old cotton seedlings. **(C)** The expression of GhCLA1 and GhPGIP1 in the control and silenced cottons were analysed by semi-quantitative RT-PCR. *GhUBQ7* was used as a reference gene. The experiments were carried out with three repeats and showed similar results.

We employed VIGS to examine the cellular function of GhPGIP1. After one month, the plants treated with pTRV1 and pTRV2-*GhPGIP1* or *GhCLA1* showed characteristic phenotype that was uniformly distributed across all true leaves (Fig. 8A,B), but the plants infiltrated with pTRV2 empty vector did not display this phenotype and grew normally.

To verify whether *GhPGIP1* has been silenced completely, total cellular RNA isolated from the plants was transcribed and the expression of *GhCLA1* and *GhPGIP1* was detected by semi-quantitative RT-PCR. The expression of *GhPGIP1* in the RNAi cotton lines (GhP1, GhP2, GhP3, and GhP4), especially in GhP1 and GhP3, was significantly lower than that in the WT plants (Ctrl; Fig. 8C). The gene *UBQ7* was used as an internal control.

### Enhanced susceptibility of GhPGIP1-silenced cotton plants to VdPG1 and FovPG1

**Figure 9:**
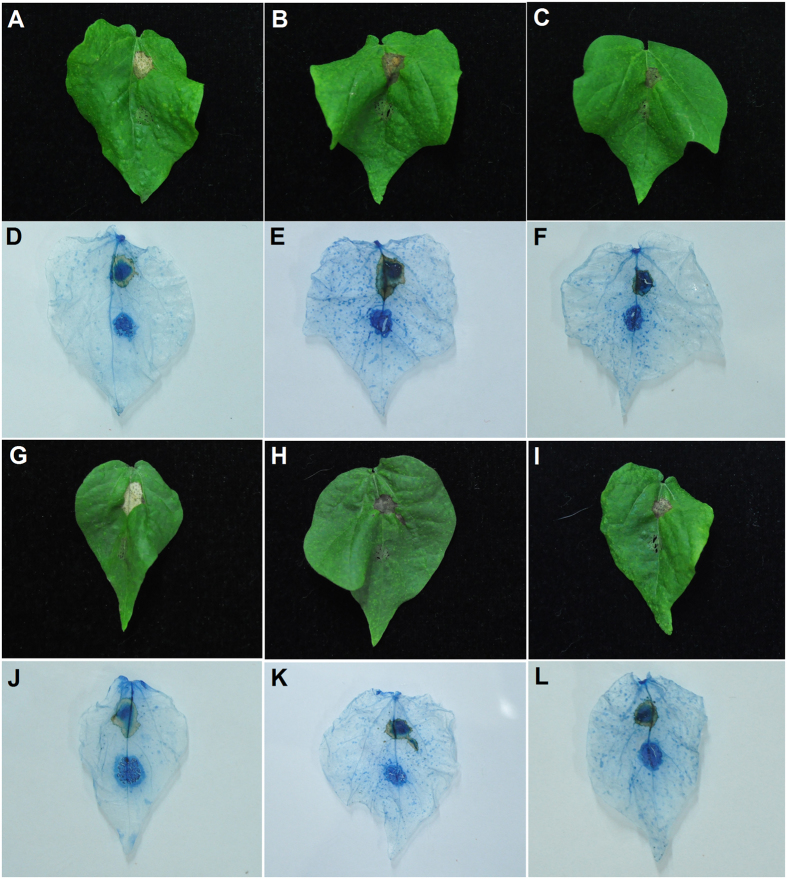
Silencing of *GhPGIP1* enhanced the cotton susceptibility to VdPG1 and FovPG1 inoculation. **(A)** Wild type cotton leaf inoculated with VdPG1 and control. **(B)** and **(C)** The GhPGIP1-silenced cotton leaves in response to VdPG1 and control. **(D)**, **(E)**, and **(F)** Trypan blue staining of the wild type (**D**) and GhPGIP1-silenced (**E**) cotton leaves, and the leaves at 5 dpi with VdPG1 (**F**). **(G)** The wild type cotton leaf inoculated with FovPG1 and control. **(H**,**I)** The GhPGIP1-silenced cotton leaves in response to FovPG1 and control. **(J**,**K**,**L)** Trypan blue staining of the wild type (**J**) and GhPGIP1-silenced (**K**) cotton leaves, and the leaves at 5 dpi with FovPG1 (**L**). The experiments were repeated three times with identical results.

To demonstrate that *GhPGIP1* is essential for resistance to *V. dahliae* and *F. oxysporum* f. sp. *vasinfectum* in cotton, four weeks after inducing GhPGIP1-silencing, WT plants were administered VdPG1 and FovPG1, respectively. The results showed that the leaves of WT cotton plants displayed a strong resistance to VdPG1 and FovPG1 (Fig. 9A,G), whereas the GhPGIP1-silenced leaves appeared to be highly susceptible, 3 d after the administration (Fig. 9B,H). Five days after treatment with the two PGs, the GhPGIP1-silenced leaves still exhibited severe disease symptoms, including larger water soaked necrotic areas, compared to the WT leaves (Fig. 9C,I). Fungal growth was analysed by trypan blue staining in the inoculated leaves. Similar to previous reports^40^, our results revealed that PG infections caused disease symptoms, with larger necrotic areas and the presence of brown lesions at the inoculation sites in the leaves compared to those in the leaves inoculated with the buffer (control) (Fig. 9D–F,J–L).

**Figure 10:**
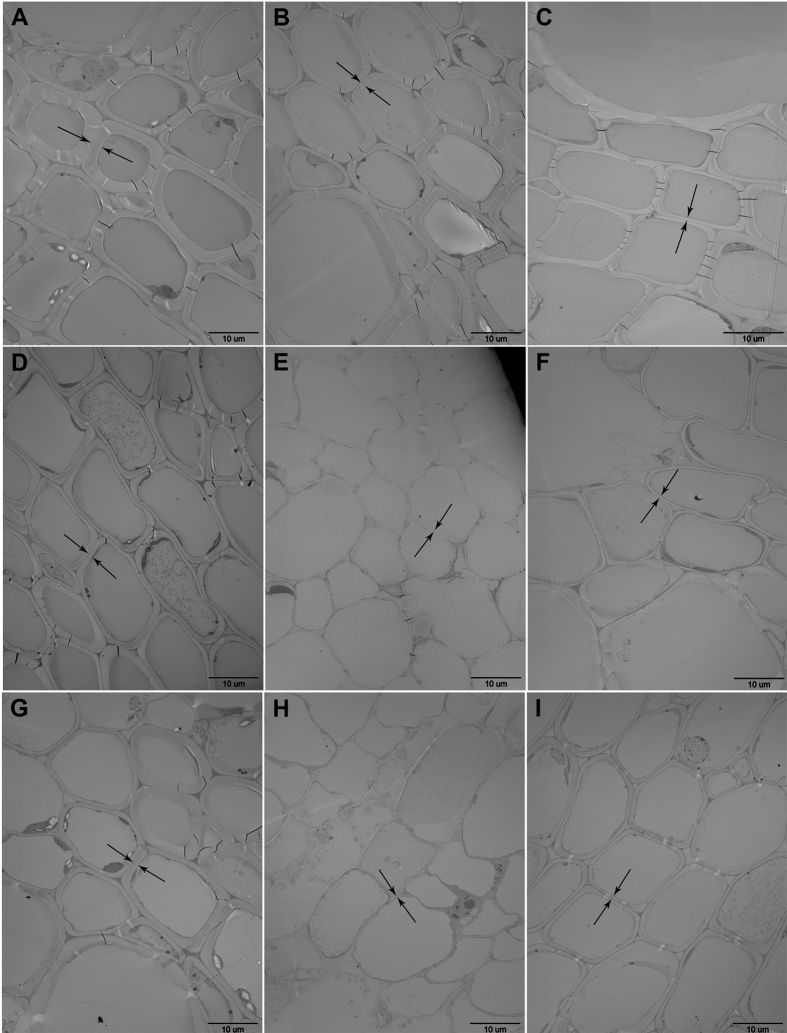
Transmission electron micrographs of VdPG1 and FovPG1 inoculated wild type and GhPGIP1-silenced cotton vascular cells. (**A**) The wild type cotton, 5 dpi with the control. (**B**) The GhPGIP1-completely silenced cotton, 5 dpi with the control. (**C**) The GhPGIP1-partially silenced cotton, 5 dpi with the control. (**D**) The wild type cotton 5 dpi with VdPG1. (**E**) The GhPGIP1-completely silenced cotton, 5 dpi with VdPG1. (**F**) The GhPGIP1-partially silenced cotton, 5 dpi with VdPG1. (**G**) The wild type cotton, 5 dpi with FovPG1. (H) The GhPGIP1-completely silenced cotton, 5 dpi with FovPG1. (**I**) The GhPGIP1-partially silenced cotton, 5 dpi with FovPG1. The black arrows indicate the position of the cell wall. Bar = 10 μm.

Inspection of the cell wall by transmission electron microscopy revealed that it was thinner in the cotton leaves treated with the two PGs than in the control leaves (Fig. 10A,B,C). After treatment with either VdPG1 or FovPG1, the cell wall in PGIP-silenced cotton plants appeared thinner than in the WT (Fig. 10D,E,F,G,H,I).

### Homology modeling

Based on the crystal structure of PvPGIP2 (1OGQ), which shares high sequence similarity with GhPGIP1, crystal structure of the latter was built. VdPG1 and FovPG1 were modelled based on the endopolygalacturonase from *Aspergillus tubingensis* (4C2L) and *Aspergillus aculeatus* (1IA5), respectively. In each case, the chosen models supported the criteria mentioned previously^13^. The final models having ideal geometry were found by MolProbity. These models were subsequently used in the docking experiments.

### Docking studies of GhPGIP1-VdPG1 and GhPGIP1-FovPG1 complexes

**Figure 11:**
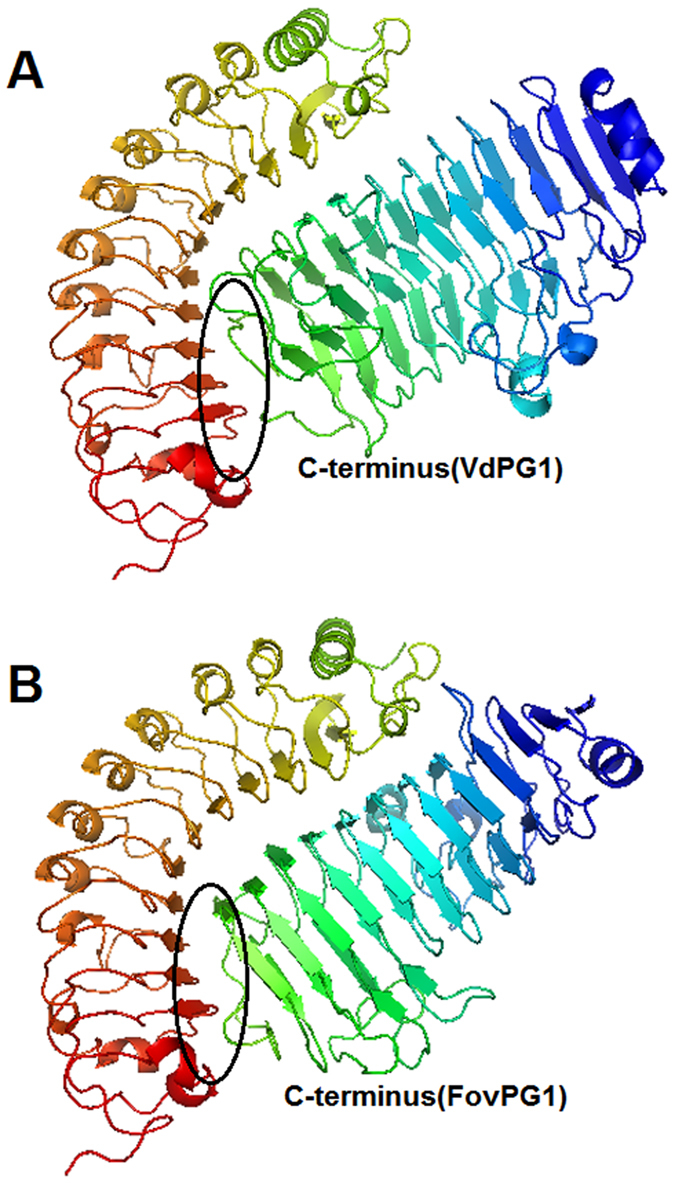
Docked complexes of GhPGIP1-VdPG1 and GhPGIP1-FovPG1. **(A)** Docked structure of GhPGIP1 and VdPG1. **(B)** Docked structure of GhPGIP1 and FovPG1. GhPGIP1 interacts through its B1-sheet with VdPG1 and FovPG1 at their C-terminus (circled in black), respectively. Most parts of the C-terminus of VdPG1 are embedded in the B1-sheet of GhPGIP1, whereas those of FovPG1 are only partially embedded.

To predict the conformation of GhPGIP1 and its putative interactions with VdPG1 and FovPG1, two different protein-protein docking programmes, PatchDock (Schneidman-Duhovny) and FireDock were used. Firstly, top 20 solutions of the complexes were generated by PatchDock, according to the scoring function used. Subsequently, FireDock was used for the fast interaction refinement of the top 10 complexes that were rescored according to the criteria described previously^41^. Then the prediction candidate models were re-ranked due to re-refinement and optimization (Supplementary Table 1). The best-docked conformations obtained in the exact expected orientations were selected and are depicted in Fig. 11A,B.

The docked protein complexes suggested that both VdPG1 (Fig. 11A) and FovPG1 (Fig. 11B) interacted with GhPGIP1 at the B1-sheet. However, the C-terminus of VdPG1 bound the B1 sheet with stronger affinity, as most parts of the C-terminus were embedded in the B1-sheet of GhPGIP1, whereas the C-terminus of FovPG1 was only partially embedded. However, the substrate-binding sites exposed in the two enzymes appeared less different (circled in black). From the docking score, the binding of GhPGIP1 with VdPG1 was predicted to be stronger than with FovPG1. The docked model, therefore, was in accordance with the experimental observation that GhPGIP1 had the ability to inhibit the enzymatic activity of VdPG1 and FovPG1.

## Discussion

In the present study, we characterized GhPGIP1 from cotton, an LRR protein classified into different families in plants. The conserved LRR domains of GhPGIP1 exhibited high similarity with the LRR regions of AtPGIP1 and AtPGIP2 (Fig. 1), with 80% similarity among them (Fig. 2). The LRR domains are conserved in all PGIPs, indicating that protein-protein interaction is involved in systemic resistance and in the recognition of non-self-molecules in plants^11^. GhPGIP1, like PGIPs of other plants, belongs to a small cluster of proteins that are individually composed of several conserved LRR domains and exhibited distinct and diverse functions^11^. These results are also supported by the notion that PGIP contains at least 10-tandemly-repeated extracellular LRR motifs^9^.

Previous reports have shown that some PGIPs are induced by biotic stress in soybean^42^, *Arabidopsis*^15^, and strawberry^43^, and might be essential for general resistance to abiotic stress^40^. In the present study, we examined the expression patterns of *GhPGIP1* in cotton after treatment with various abiotic and biotic stresses. Our results revealed that *GhPGIP1* was significantly induced by *V. dahliae* and *F. oxysporum* f. sp. *vasinfectum* infection on 3 and 7 d, respectively (Fig. 3A,B), providing protection against the diseases. The observed expression of *GhPGIP1* at different times, post MeJA treatment (Fig. 3C), was different from that reported for *CaPGIP* in *C. annuum*^18^. In *Arabidopsis*^44^ and bean^20^, PGIPs accumulated in response to SA treatment, providing disease resistance. In our study, SA treatment induced the up-regulation of *GhPGIP1*, and its expression peaked at 6 h (Fig. 3D), similar to that of *CaPGIP* in *C. annuum*^18^. Hydrogen peroxide plays a central role in launching the defence response during stress in plants^45^; our study showed increased transcription of *GhPGIP1* with a peak at 6 h (Fig. 3E). Additionally, wounding can induce defence proteins to provide protection from stresses^15^. In our study, wounding induced up-regulation of *GhPGIP1* (Fig. 3F). These findings suggest that plants induce sophisticated defence mechanisms to regulate their responses when interacting with the surrounding environment.

The optimum pH for the inhibition of *Aspergillus niger* PG by PGIP-1 and PGIP-2 from guava fruit was determined to be 4.2, whereas that for PGIP-3 was 4.4, when assayed over a pH range of 4.4–4.5^46^. However, PGIP from chilli retained >50% activity at pH 3.0 and 8.0^47^. Previous studies on thermal tolerance of PGIPs have shown them to be thermostable. PGIPs isolated from orange retained considerable activity at 60 °C^48^, and that from tomato retained partial inhibitory activity at 100 °C^49^. In this study, GhPGIP1 inhibited VdPG1 effectively with an inhibition rate of 26.88–31.45% over a pH range of 4.2–4.8, and inhibited FovPG1 over a pH range of 8.0–9.0, with an inhibition rate of 23.79–29.61% (Fig. 5B,C). GhPGIP1 remained active from 20 °C to 60 °C; the activity dropped from 70 °C to 90 °C (Fig. 5D), which was consistent with the results of a previous study^50^. This diversity in PGIP activity at different pH and temperature could be crucial in countering the multitude of PGs encountered from various pathogens. Such differential PGIP activities have been proposed to generate a steady-state concentration of biologically active oligogalacturonides that participate in producing a better host defence response^51^.

To determine the effect of gain-of-function of GhPGIP1 in plants, we generated GhPGIP1 transgenic *Arabidopsis* lines and examined their response to *V. dahliae* and *F. oxysporum* f. sp. *vasinfectum*; these pathogens can cause yellowing, wilting, defoliation, and eventual death in cotton plants^52^. *Arabidopsis* was chosen for the ease of its transformation. The qRT-PCR analysis revealed high expression levels of GhPGIP1 in the different transgenic lines (Supplementary Fig. 3). Moreover, the results revealed that the accumulation of GhPGIP1 in transgenic *Arabidopsis* plants could endow them with enhanced inhibition capabilities against PGs, reducing the disease symptoms in transgenic plants challenged with the two fungal pathogens (Fig. 6). This provides indirect evidence that PGs are important pathogenic factors in the infection of *Arabidopsis* by the two fungal pathogens. Previous studies have reported the effectiveness of PGIP in reducing the disease symptoms in other transgenic plants infected with *B. cinerea* and *Cercospora nicotianae*^18^,^41^. Here, we provide evidence that GhPGIP1 gene can also protect *Arabidopsis* against infection by the two fungi. The significant reduction in the lesion size indicates that GhPGIP1 might inhibit the PGs from the two pathogens during the formation of initial lesion as well as during the expansion of the lesion.

To counter the threat posed by pathogens, plants have evolved several defence mechanisms that include the expression of a large number of defence genes. Transgenic expression of defence genes in plants can help in better understanding of the molecular mechanisms involved in the response of plants to pathogens stress. The toxin of *V. dahliae* has been demonstrated to possess dual functions: that of a toxin and an elicitor, at high and low concentrations, respectively^53^,^54^. Numerous studies have shown that SA plays a vital role in defence signalling pathways and its levels increase with the activation of PR gene^55^. Plants are believed to reserve SA as glucoside SA, which releases free SA under the action of SA GTase, thereby, inducing the PR gene expression. ICS1 is an essential enzyme in pathogen-induced biosynthesis of SA and is necessary for understanding the SA-mediated defence responses^56^,^57^. EDS1-regulated, SA-antagonized, and SA-promoted processes are indispensable for resistance to pathogens. *EDS1* regulates SA accumulation by elevating its own expression and that of other genes^39^. For instance, *EDS1* is indispensable for the accumulation of pathogen-induced *PAD4*. In the *EDS1*/*PAD4*-dependent R gene-mediated response, both these genes positively regulate the accumulation of SA. Furthermore, in the induction of plant hypersensitive response (HR), *EDS1* is especially necessary^58^,^59^. Our experiments demonstrated that all the SA-responsive genes were up-regulated during *V. dahliae* infection (Fig. 7), suggesting that GhPGIP1 contributes to the SA-mediated defence responses and plays a positive role in the regulation of gene expression.

We used VIGS approach to investigate the effect of loss of function of *GhPGIP1* in cotton infected with highly virulent strains of *V. dahliae* and *F. oxysporum* f. sp. *vasinfectum*. The control plants showed relatively high *GhPGIP1* expression, whereas various treatments caused a decrease in its transcript levels in PGIP-silenced cotton (Fig. 8). Moreover, PGIP-silenced cotton exhibited enhanced susceptibility to the purified PGs (Fig. 9B,C,H,I) and the control cotton showed enhanced resistance to the two PGs (Fig. 9A,G). Therefore, we concluded that basal disease resistance of cotton was possibly activated on exposure with the two pathogens, and GhPGIP1 might be participating in inhibition at different stages of the infection. To further investigate the cell wall alterations of the vascular elements, the two PGs purified from the two vascular fungi were inoculated in the control and PGIP-silenced cotton. The results showed thinner cell walls after inoculation with both the PGs, compared to the control; however, the PGIP-silenced cotton revealed thinner cell walls than the control cotton (Fig. 10). The marked decrease in thickness of the cell walls in PGIP-silenced cotton suggested that PGs mainly degrade cell walls, and can be effectively inhibited by PGIP; this adversely affects the growth and development of the pathogen protects the cell wall integrity. These observations provide indirect evidence for the role of GhPGIP1 in protecting the plant cell walls against fungal pathogens.

The PG-PGIP interaction, considered a model protein-protein recognition in plant-pathogen interactions^60^, is paradigmatic for studying the key recognition events underlying plant immunity^60^,^61^. The concave face of PGIPs participates in establishing the specificity for binding to the invading PG, whereas the convex face imparts the required flexibility^13^. To further examine the interaction between GhPGIP1 and the two PGs, docking studies were performed. The results indicated remarkable differences between the docking complexes, reflected in the interaction modes of enzyme-inhibitor complexes in Fig. 11. GhPGIP1 was observed to contact VdPG1 only at the C-terminal of B1-sheet, in the VdPG1-GhPGIP1 complex; the other parts of VdPG1 were still accessible to the substrate, which had the potential to form ternary complexes with GhPGIP1 (Fig. 11A). In the FovPG1-GhPGIP1 complex, most part of the active site was almost covered by the inhibitor (Fig. 11B). These studies demonstrated the structural flexibility and specificity of GhPGIP1 binding interactions with the two PGs.

In conclusion, we have demonstrated that transgenic *Arabidopsis* plants overexpressing GhPGIP1 were more resistant to *V. dahliae* and *F. oxysporum* f. sp. *vasinfectum*, and conversely, cotton with partially silenced GhPGIP1 expression was more sensitive to VdPG1 and FovPG1 infection. Moreover, we investigated the interaction between GhPGIP1 and the two PGs, which enhanced our understanding of this interaction at the structure level. However, structural analyses would be necessary for drawing structure-function correlation. Furthermore, the multiplicity of GhPGIP1 functions would be crucial for screening host proteins with improved antifungal potential against the evolving PGs.

## Methods

### Plant growth and preparation of fungal polygalacturonases

The state cotton 2006001 (original strain no. GK44), was provided by the Cotton Research Institute, Chinese Academy of Agricultural Sciences and its seedlings were used for inoculation with the two fungi. RNA was extracted from the inoculated seedlings for gene expression analysis and for the preparation of cDNA library. The virulent strains of *Verticillium dahliae* Vd991 and *Fusarium oxysporum* f. sp. *vasinfectum* were cultured on potato dextrose agar on cellophane sheets for 7 d. New hyphae were collected and used for the extraction of RNA with Trizol; the extracted RNA was subsequently reverse transcribed.

### RNA extraction and cloning of *GhPGIP1*

Total RNA was extracted from the cotton plants according to the manufacturer’s instructions (Promega, WI, USA). A PolyATract mRNA Isolation System was used to obtain polyadenylated mRNA following the specifications of the supplier (Promega). Thereafter, a cDNA library was constructed as described previously^41^,^62^, based on a 21-amino acid sequence (5′-FDXSYFHNKCLCGAPLPSCK-3′) conserved at the C-terminus of all the previously characterized PGIPs^9^,^15^.

### Sequence manipulation and phylogenetic analyses

A homology search against the NCBI database was conducted to verify whether the obtained sequences encoded GhPGIP1. Multiple protein sequence alignment was performed using Clustal Omega (http://www.ebi.ac.uk/Tools/msa/clustalo/) and the software SMART (http://smart.embl-heidelberg.de/) was used for the prediction of domain structure. A phylogenetic tree based on the protein sequences was constructed using MEGA 5.1.

### RNA isolation and qRT-PCR analysis of plants treated with various stresses

The cotton seedlings were grown at day/night temperatures of 25 °C/22 °C in a growth chamber under a light intensity of 150 μmol·m^−2^·s^−1^ provided during a 16/8-h photoperiod. Total RNA was extracted (Biomed) from the complete stool of cotton plants that were exposed to one of the following treatments for induction of stress response: infliction with wounds, administration of 5 mM salicylic acid^18^, 100 μM methyl jasmonate (MeJA) (Sigma, St. Louis)^63^, or 10 mM H_2_O_2_^64^, or infection with *V. dahliae* or *F. oxysporum* f. sp. *vasinfectum*^65^. The total RNA was reverse transcribed (TIANGEN BIOTECH CO., LTD, Beijing, China). The qRT-PCR analysis for GhPGIP1 was performed using specific primers, qGhPGIP-F: 5′-TCTGGTACAATCCCTGCCTC-3′ and qGhPGIP-R: 5′-CAGATCCAGCCTTGCCAAAC-3′; the endogenous control used was *UBQ7* gene (DQ116441) from cotton, which was detected using the sense primer UBQ-F: 5′-GAAGGCATTCCACCTGACCAAC-3′ and antisense primer UBQ-R: 5′-CTTGACCTTCTTCTTCTTGTGCTTG-3′. The transcription levels of the target gene (*GhPGIP1*) relative to the reference gene (*UBQ7*) were analysed by the comparative CT (2^−ΔΔCT^) method, where ΔΔCT = (C_T_ target-C_T_ reference)_Sample X_ − (C_T_ target-C_T_ reference)_Sample 1_. Sample 1 was the calibrator sample without any treatment and Sample X represents the samples from the plants exposed to the different stress treatments. All the experiments were repeated three times from three independent plants. The data were analysed statistically using SPSS (IBM Corp., Armonk, NY). The data analyses were performed by one-way analysis of variance (ANOVA). The asterisk indicates a significant difference at *P* < 0.5, based on the least significant difference (LSD) method.

### Expression and purification of recombinant GhPGIP1 protein

*GhPGIP1* was amplified using the primers 5′-CTACATATGgACCACTgCAACgCTCAAgACAAg -3′ and 5′-TAAGGATCCTTATTACTTgCAgACgTCgAgCggAg-3′. The amplified fragment was subsequently digested with NdeI and BamHI, and cloned into PET-32a vector with a six-His tag. The construct was transformed into *Escherichia coli* BL21DE3 following the protocol of the manufacturer for competent BL21 DE3 cells (CW BIO, Beijing, China). The single transformed colonies obtained were cultured at 37 °C until the optical density reached 0.6 to 0.8, and the cells were then induced with 1.0 mM IPTG. The culture was further incubated for 6 h at 28 °C and was harvested, thereafter, by centrifugation at 10,000 × *g* for 20 min at 28 °C. The expression of soluble GhPGIP1 was verified by sodium dodecyl sulfate polyacrylamide gel electrophoresis (SDS-PAGE) and the recombinant protein was purified using a 6× His-Tagged Protein Purification Kit (CW BIO)^41^. The 6× His fusion tags, trx∙tag and S∙tag, were removed using a Thrombin Cleavage Capture Kit (Novagen, Madison, WI).

### Inhibitory activity of GhPGIP1 and agarose diffusion experiment

The GhPGIP1 activity was determined by reducing the end groups with a modified DNS reagent^66^. The plate assay was performed using a reaction mixture containing 0.8% agarose and 0.5% polygalacturonic acid^18^. VdPG1 and FovPG1 were added with or without GhPGIP1 on the agarose plates, the mixture was incubated at 30 °C for 12 h, and then 0.05% ruthenium red was used to stain the agarose plates, followed by thorough washing with sterile water^66^. The PG activity was expressed as agarose diffusion units, with a ring of 0.5 cm radius defined as one agarose diffusion unit. One unit activity of PGIP was defined as the amount of PGIP required to reduce the PG activity by 50%^18^. The GhPGIP1 mixture containing 0.0011 reducing units was used to assess the inhibitory activity of GhPGIP1.

### PGIP activity measurements

The purified VdPG1 (15 ng) and FovPG1 (25 ng) were incubated separately with 0.1 mg/mL of polygalacturonic acid substrate (Sigma) at 30 °C in 50 mM sodium acetate buffer (pH 4.8 and pH 8.9, respectively), in reaction volumes of 200 μL^50^. The PG activity was determined by analysis of the reducing end-group according to Anthon and Barrett^67^. The activity of the PGs, pre-incubated with GhPGIP1, for 20 min at 30 °C, was used to assay the inhibitory activity of PGIP. The inhibitory activity was expressed as percentage reduction in the number of reducing ends (in μkat/mg of protein) liberated by PGs in the presence of PGIP^50^. The PGs pre-incubated with VC served as the control.

In separate experiments, the temperature and pH stability of GhPGIP1 were studied by pre-incubating the GhPGIP1 solutions separately for 1 h at temperatures ranging from 20 to 100 °C, and for 16 h at pH ranging from 2.0 to 11.0 at 4 °C, respectively. The pre-incubated GhPGIP1 solutions were reconstituted in the appropriate assay buffer and their inhibition potential was assayed at 30 °C^50^. All the experiments were performed twice, each time in triplicates. The data of a representative experiment was subjected to Tukey’s Honestly Significant Difference (HSD) test followed by analysis of variance at *P* < 0.05.

### Disease assays and lesion-size determination in transgenic *Arabidopsis* plants

*GhPGIP1*was amplified using the forward (5′-gCCAAgCTTATgAAgATATATCCAgCTTTCCT-3′) and reverse (5′-ATATAAACTAgTCTTgCAgACgTCgAgCggAg-3′) primers, which incorporated *Hind*III and *Spe*I cleavage sites, respectively. The amplified gene was subcloned into Super-pCAMBIA1300 (Supplementary Fig. 4). *V. dahliae* (Vd991) and *F. oxysporum* f. sp. *vasinfectum* were grown on potato dextrose agar at room temperature (23 °C) for 3–4 d. The spore suspensions were prepared as described elsewhere^21^. The wild type and transgenic *Arabidopsis* plants were grown for 5 weeks in growth chambers at 24 °C under conditions of 70% relative humidity and 16 h light/8 h dark cycle. The plants were then challenged with a conidial suspension of *V. dahliae* and *F. oxysporum* f. sp. *vasinfectum* by syringe inoculation as reported earlier^41^. The disease lesion areas were measured after 3–5 d. All the disease assays were repeated at least four times.

### Histochemical staining and cell death quantification

The leaves from transgenic (line 2 and line 6) and wild-type *Arabidopsis* plants inoculated with *V. dahliae* and *F. oxysporum* f. sp. *vasinfectum* were collected for histochemical staining. Lactophenol trypan blue was used to detect the dead or dying cells as described elsewhere^39^,^68^. To identify the dead cells, the inoculation spots (1 mm^2^) were marked on the leaves, as reported previously^39^. The accumulation of H_2_O_2_ was detected following the protocol of Torres^69^. Briefly, the leaves were infiltrated with 3–3′-diaminobenzidine (Sigma) solution and placed in a lucifugal beaker for 8–10 h, and then dipped in an ethanol/lactic acid/glycerol solution (3:1:1) for 6–8 h, followed by decolouration with chloral hydrate^39^ (Sigma). The stained leaves were then observed by an imaging system and the photographs were taken with a Nikon Eclipse Ti microscope (Japan) using a 4 × 0.25 numerical aperture objective.

### RNA extraction, reverse transcription, and qRT-PCR

RNA was isolated from the complete stools of wild-type and transgenic *Arabidopsis* plants inoculated with *V. dahliae*, using Trizol reagent (TIANGEN BIOTECH CO., LTD, Beijing, China) as per the manufacturer’s instructions. Total RNA was reverse transcribed by FastQuant RT Kit (with gDNase; TIANGEN BIOTECH CO., LTD, Beijing, China). qPCR was performed with a SYBR^®^ Premix Ex Taq (Takara, Japan) according to the manufacturer’s protocol using gene specific primers; *Arabidopsis EF-1-α* was used as an endogenous reference. The qPCR assays were performed for at least three biological replicates and a minimum of three technical replicates for each sample. The primers used are listed in Supplementary Table 2.

### Construction of virus-induced gene silencing vector and semi-quantitative RT-PCR

Fragments of *GhPGIP1* (491 bp) and *GhCLA1 (Cloroplastos alterados* 1; 500 bp) were PCR amplified from the cotton cDNA and inserted into an improved pTRV2 VIGS vector, pYL192^70^,^71^. The primer sequences used were 5′-CGACGACAAGACCGTGACCATGCACAACATCGATGATTTAG-3′ (GhCLA1-F), 5′-GAGGAGAAGAGCCGTCATTAGCATGAATGATGAGTAGATTGCAC-3′ (GhCLA1-R), 5′-CGACGACAAGACCGTGACCATGAACTTAAGAACTTGACTTAC-3′ (GhPGIP1-F), and 5′- GAGGAGAAGAGCCGTCATTAGAACTGCAACTCCAGATCCG-3′ (GhPGIP1-R). The two products were cloned into pTRV2 (a kind gift from Professor Yule Liu, Tsinghua University, China) to construct pTRV1- pTRV2: GhCLA1 and pTRV1- pTRV2: GhPGIP1. The plasmids containing the binary TRV vectors were transformed into *A. tumefaciens* strain GV3101. The culture was grown overnight at 28 °C in LB medium containing 50 μg/mL kanamycin, 50 μg/mL rifampicin, 10 mM MES, and 20 μM acetosyringone; the cells were handled as described earlier^21^. The *Agrobacterium* cultures containing pTRV1 and pTRV2: GhCLA1or pTRV2: GhPGIP1 were mixed in a 1:1 ratio and the fully extended cotyledons of 2-week-old cotton seedlings were co-infiltrated with a needle-less syringe. To facilitate the infiltration, small holes were punched on the underside of the cotyledon using a needle^21^. The assays were performed with at least six plants for each construct and the experiments were repeated at least thrice.

The expression of GhCLA1 and GhPGIP1 in the VIGS silenced-lines was detected in the detached cotton leaves using semi-quantitative RT-PCR. The leaves were collected five weeks after *Agrobacterium* infiltration and subjected to RNA extraction using an RNA Extraction Kit (Biomed, Beijing, China), and the total RNA was reverse transcribed (TIANGEN BIOTECH CO., LTD, Beijing, China). The *GhUBQ7* gene was used as the internal control. The primers used in the RT-PCR analysis are listed in Supplementary Table 2.

### VdPG1 and FovPG1 infiltration of cotton plants

Infiltrations were performed with a needle-less 1-mL or 2-mL syringe placed against the upper side of the leaves^72^; the lower side of the leaves were inoculated with the respective PG dissolutions and treated with a bacterial lysis buffer, followed by binding, and were subsequently treated with an elution buffer^18^. The solutions of VdPG1 and FovPG1 in sodium acetate, pH 4.8 and pH 8.9, respectively, were applied in varying concentrations. Before infiltration, the enzyme activities were determined using 0.25% (w/v) polygalacturonic acid, as described previously^72^. In each experiment, the PG and control solutions were inoculated in two sections per leaf, two leaves per plant, and more than three plants for each treatment. The experiments were performed at least thrice with different batches of the enzymes. Trypan blue staining was performed as mentioned above.

### Homology modelling of GhPGIP1, VdPG1, and FovPG1

The X-ray crystallographic structures of PGIP2 (PDB ID: 1OGQ), enzymes from *Aspergillus tubingensis* (PDB ID: 4C2L), and *Aspergillus aculeatus* (PDB ID: 1IA5) were used as models for GhPGIP1 (Supplementary Fig. 5A), and polygalacturonases from *V. dahliae* (Supplementary Fig. 5B) and *F. oxysporum* f. sp. *vasinfectum* (Supplementary Fig. 5C), respectively. The structures were modelled using SWISS-MODEL^41^. The post-refinement of the structure models was carried out using the KoBaMIN server (Rodrigues), and further geometric accuracy of the models was evaluated using MolProbity 4.02b^73^. The final structures were visualised in Pymol.

### Docking studies

We used PatchDock (Schneidman-Duhovny) beta 1.3 version molecular docking algorithm based on shape complementarity principles for docking GhPGIP1 to the two PGs, individually. For the protein–protein interactions, the receptor molecule was GhPGIP1 and the ligand molecules were VdPG1 and FovPG1. The output of the analysis contained 20 models with the highest-scoring ones being the most probable^13^. To select the most suitable model from among the top-20 docked complexes, the output was resubmitted to FiberDock for further optimization and re-refinement.

## Additional Information

**How to cite this article**: Liu, N. *et al*. Molecular evidence for the involvement of a polygalacturonase-inhibiting protein, GhPGIP1, in enhanced resistance to Verticillium and Fusarium wilts in cotton. *Sci. Rep.* **7**, 39840; doi: 10.1038/srep39840 (2017).

**Publisher's note:** Springer Nature remains neutral with regard to jurisdictional claims in published maps and institutional affiliations.

## Supplementary Material

Supplementary Information

## Figures and Tables

**Table 1:** Effect of GhPGIP1 on the growth of fungal pathogens.

Pathogen	IC_50_ (μg/mL)
*V. dahliae*	44.37
*Fusarium* f. sp. *vasinfectum*	50.13
*B. cinerea*	55.24
*Valsa mali*	90.66
*Rhizoctonia solani*	110.35

Eight doses of the purified GhPGIP1 were prepared for the measurement of antifungal activity (half maximal inhibitory concentration, IC_50_). The buffer was used as the control.
