# Supplementary material for: Molecular evidence for the involvement of a polygalacturonase-inhibiting protein, GhPGIP1, in enhanced resistance to Verticillium and Fusarium wilts in cotton
Source: Sci Rep. 2017 Jan 12;7:39840. doi: 10.1038/srep39840 (PMC5228132; doi:10.1038/srep39840)
Supplement: Supplementary Information [file srep39840-s1.pdf]

# Molecular evidence for the involvement of a polygalacturonase-inhibiting protein, GhPGIP1, in enhanced resistance to Verticillium and Fusarium wilts in cotton

Nana Liu[1], Xueyan Zhang[2], Yun Sun[1], Ping Wang[1], Xiancai Li[1], Yakun Pei[1]
Fuguang Li[2]*&Yuxia Hou[1]*

[1]College of Science, China Agricultural University, No. 2 Yuanmingyuan West Road, Beijing 100193, People's Republic of China. [2]State Key Laboratory of Cotton Biology, Institute of Cotton Research of the Chinese Academy of Agricultural Sciences, Anyang 455000, People's Republic of China. Correspondence and requests for materials should be addressed to F.L. (email: aylifug@163.com) or Y.H. (email: houyuxia@cau.edu.cn)

Compliance with ethical standards: The study described in this article did not involve experimentation on humans and animals, and the plant species used in the study is not on the verge of extinction.

```
1    ATGAAGATATATCCAGCTTTCCTCAGTTTCCTCTTCATATCCATCTTCATTTCACCTTCT
      M  K  I  Y  P  A  F  L  S  F  L  F  I  S  I  F  I  S  P  S    20

61   GTCTCAGACCACTGCAACGCTCAAGACAAGAAGGTTCTTCTCAAGATCAAAAAGGCCTTA
      V  S  D  H  C  N  A  Q  D  K  K  V  L  L  K  I  K  K  A  L    40

121  GGCAACCCTTACCTCTTGGCCTCATGGTACCCCAACACTGATTGCTGTGATTGGTACTGT
      G  N  P  Y  L  L  A  S  W  Y  P  N  T  D  C  C  D  W  Y  C    60

181  CTTGAATGCCATCCCAATACCCACCGTGTCGTTTCCCTCACCCTGTTCTCCGATGATCGT
      L  E  C  H  P  N  T  H  R  V  V  S  L  T  L  F  S  D  D  R    80

241  CTCACCGGCCAAATCCCCCCTGAAGTTGGCGACCTTCCGTACCTTGAAGCCCTTCTTTTC
      L  T  G  Q  I  P  P  E  V  G  D  L  P  Y  L  E  A  L  L  F    100

301  AGACACCTCCCTAACCTCAATGGAACCATACAACCCGCCATTGCCAAGCTCAAGAACCTC
      R  H  L  P  N  L  N  G  T  I  Q  P  A  I  A  K  L  K  N  L    120

361  AAGACGCTTCGCTTGAGCTGGACCAATCTTTCCGGTCCAGTCCCTAATTTTCTTAGCCAA
      K  T  L  R  L  S  W  T  N  L  S  G  P  V  P  N  F  L  S  Q    140

421  CTTAAGAACTTGACTTACTTGGACCTTTCATTTAATAACCTCTCGGGATCTATTCCAAGC
      L  K  N  L  T  Y  L  D  L  S  F  N  N  L  S  G  S  I  P  S    160

481  TCCCTTTCAACACTCCCAAATCTCGAGGCTTTGCATTTGGATAGAAACAAGTTAACTGGT
      S  L  S  T  L  P  N  L  E  A  L  H  L  D  R  N  K  L  T  G    180

541  ACCATACCAGAATCTTTTGGTATGTTTCCTAGCAAAAACCTGTATTTGTTCATCTTGTCT
      T  I  P  E  S  F  G  M  F  P  S  K  N  L  Y  L  F  I  L  S    200

601  CACAATAAACTTTCTGGTACAATCCCTGCCTCTTTAGCCAACATGGACTTTAACACCATT
      H  N  K  L  S  G  T  I  P  A  S  L  A  N  M  D  F  N  T  I    220

661  GACCTGTCGAGGAACTTGCTTGAAGGTGATCCTTCAGTGTTGTTTGGCCCGAAAAAAACG
      D  L  S  R  N  L  L  E  G  D  P  S  V  L  F  G  P  K  K  T    240

721  ACATTCGAAATCGATCTTTCTAGGAACATGTTCCAATTCGATCTATCTAAAGTGCAGTTT
      T  F  E  I  D  L  S  R  N  M  F  Q  F  D  L  S  K  V  Q  F    260

781  CCCAAGAGTTTGGCAAGGCTGGATCTGAATCATAACAAGATTACGGGAAGTATTCCTGCA
      P  K  S  L  A  R  L  D  L  N  H  N  K  I  T  G  S  I  P  A    280

841  GGGTTGACGGATCTGGAGTTGCAGTTCATGAATGTAAGTTACAATAGGTTGTGCGGACAA
      G  L  T  D  L  E  L  Q  F  M  N  V  S  Y  N  R  L  C  G  Q    300

901  ATCCCGGTGGGAGGGCGGTTGCAGAGTTTCGATTACTCCACGTATTTTCACAACCGCTGC
      I  P  V  G  G  R  L  Q  S  F  D  Y  S  T  Y  F  H  N  R  C    320

961  TTGTGCGGTGCTCCGCTCGACGTCTGCAAGTAAAGGTGCGCTGCCGACATTATTAAGTTA
      L  C  G  A  P  L  D  V  C  K  *                                330

1021GGGCTTTCATCATGCATCAAAGTGCAGGTTGGAGAGTTGTAATAAGCACGCACATGTTTT

1081TATTTTGACTAAGCTATTCAGGGAAAATATCTTTTTATCAGCAATTTGAGAATAAACGAC

1141ATTGTATTTCACCCAAGAATTAGTGAACTTTACAAACACCTAAAAAAAAAAAA
```

**Supplementary Figure 1. cDNA and amino acid sequence of *GhPGIP1* from *Gossypium hirsutum*.** The signal peptide and N-glycosylation sites are highlighted in green with gray shadow and yellow, respectively. LRRNT-2 and LRR domains are singly and doubly underlined, respectively. Cysteines are marked with red.

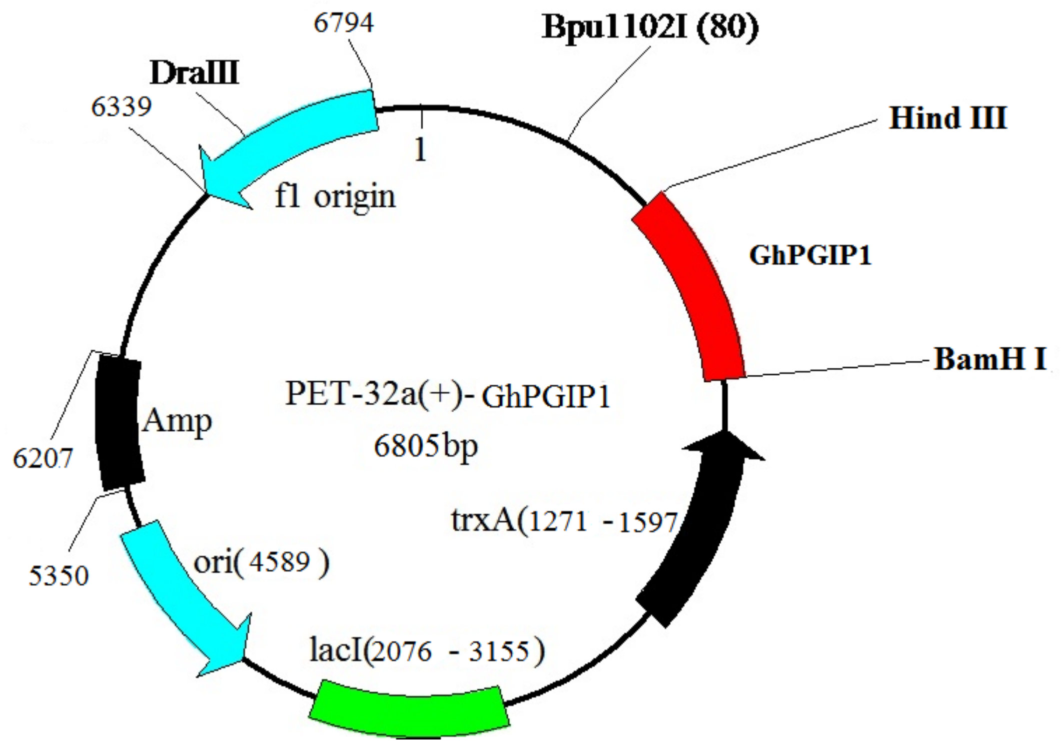


**Supplementary Figure 2. Schematic representation of PET-32a-CkPGIP1 bacterial expression plasmid.**

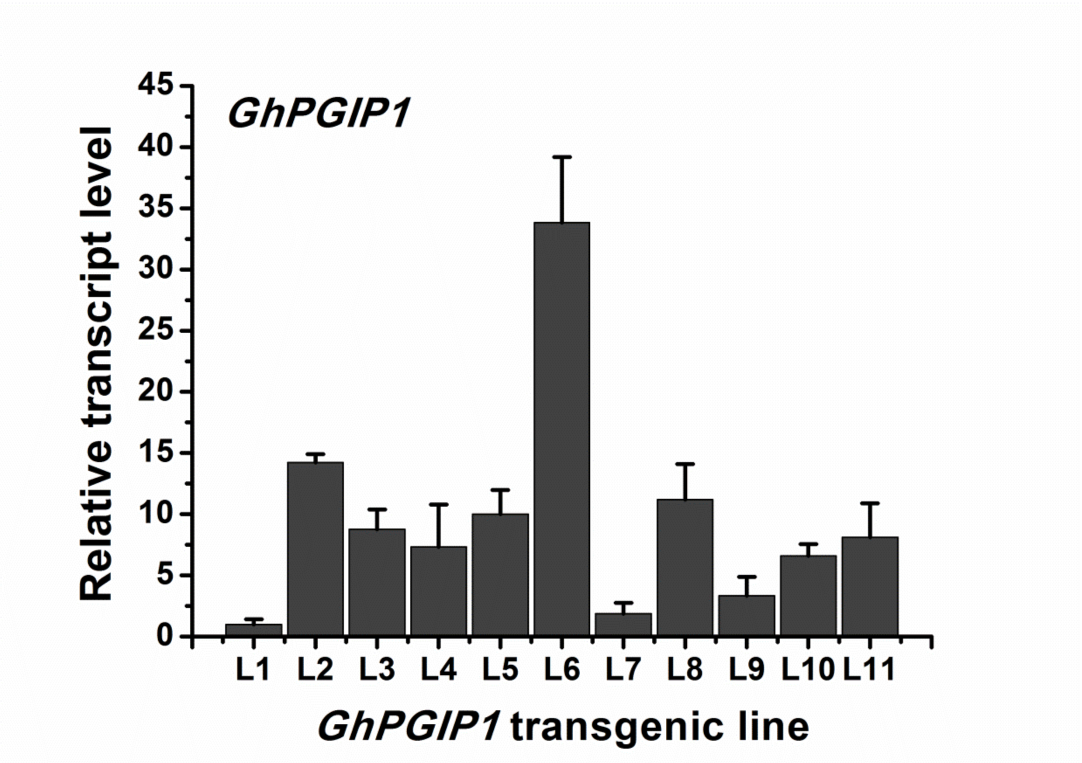


**Supplementary Figure 3. Selection and transcript level identification in transgenic Arabidopsis.** Lines 1–11 were genotyped by PCR, and 11 were found to be transgenic. Real-time PCR was then used to measure relative expression of *GhPGIP1*. The experiment was carried out with three biological replicates.

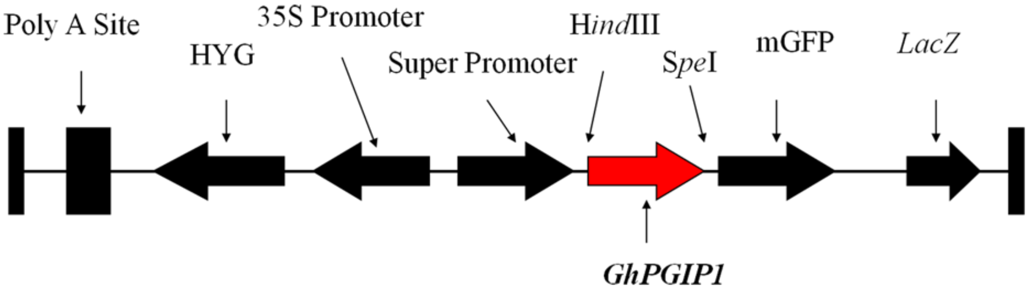


**Supplementary Figure 4. Pictorial representation of the plant expression vector pCAMBIA1300-CkPGIP1.** H*ind*III and S*pe*I are the double digestion of restriction sites.

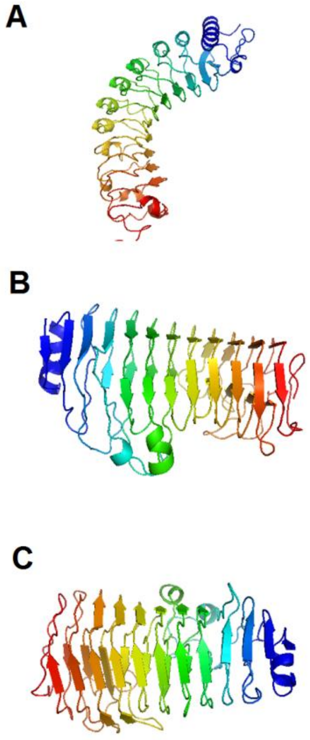

**Supplementary Figure 5. Homology modeling.** Homology models of GhPGIP1 (**A**), *Verticillium dahliae* (**B**), and *Fusarium oxysporum* f. sp. *vasinfectum* polygalacturonase (**C**) are based on known structures of PvPGIP (1OGQ), and polygalacturonase from *Aspergillus Tubingensis* (4C2L) and *Aspergillus aculeatus* (1IA5).

| | GhPGIP-VdPG1(B) | GhPGIP-VdPG1(A) | GhPGIP-FovPG1 (B) | GhPGIP-FovPG1 (A) |
|---|---|---|---|---|
| glob | 11.05 | -12.58 | 10.08 | 1.93 |
| aVdW | -37.72 | -28.30 | -31.49 | -21.71 |
| rVdW | 27.22 | 17.47 | 10.09 | 1.99 |
| ACE | 14.30 | 8.28 | 15.62 | 10.62 |
| HB | -2.47 | -3.06 | -1.78 | -4.84 |

**Supplementary Table 1. The comparison of all the changes about the complexes before and after optimization.**(**A**): before optimization;(**B**): after optimization. glob: the binding energy of the solution ; aVdWa and rVdW :softened attractive and repulsive van der Waals energy; ACE: atomic contact energy; HB: hydrogen and disulfide bonds.

| Primer name | Primer sequences |
|---|---|
| qEF1α-F | 5′-TCAGGAAGCTCTTCCTGGTG-3′ |
| qEF1α-R | 5′-CAATGTGAGAGGTGTGGCAG-3′ |
| qPR1-F | 5′-CATACACTCTGGTGGGCCTT-3′ |
| qPR1-R | 5′-CTCGGATGTGCCAAAGTGAG-3′ |
| qPR5-F | 5′-TCACTCTAGTAGGCGATGGCG-3′ |
| qPR5-R | 5′-GCAGGCCACGACATTGTTCTG-3′ |
| qICS1-F | 5′- CGAGAACGGAAACGGAAACG-3′ |
| qICS1-R | 5′- GCATACCACCATAGGCACGA-3′ |
| qEDS1-F | 5′- GGAAGGGCTTCCACTCTCGT-3′ |
| qEDS1-R | 5′- GCCCACCTTGCACAATGCAG-3′ |
| qPAD4-F | 5′- CGACCTCGTTCCTAGAAGCA-3′ |
| qPAD4-R | 5′- TGAACACATAGTGTCCGTACCT-3′ |
| qPDF1.2-F | 5′- TCATCACCCTTATCTTCGCTG-3′ |
| qPDF1.2-R | 5′- ATGTCCCACTTGGCTTCTC-3′ |
| GhP1-F | 5′- CCAAGCTCCCTTTCAACACT-3′ |
| GhP1-R | 5′- CCTTCAAGCAAGTTCCTCGACA-3′ |
| GhC1-F | 5′- CCGAGGTTATCCATATGCAGAG-3′ |
| GhC1-R | 5′- GGCGGAGGAAGAGGTTTAA-3′ |
| GhUBQ7-F | 5’-GAAGGCATTCCACCTGACCAAC-3’ |
| GhUBQ7-R | 5’-CTTGACCTTCTTCTTCTTGTGCTTG-3’ |

**Supplementary Table 2.** Primer names and sequences. qRT-PCR, the primer sequences used are summarized in the table.
